# Effects of the online and offline hybrid continuous group care on maternal and infant health: a randomized controlled trial

**DOI:** 10.1186/s12884-023-05882-1

**Published:** 2023-09-01

**Authors:** Xiaoli Yang, Linwei Li, Rong Zhou, Jieqiong Xia, Minxiang Li, Caihong Zhang, Honghua Guo

**Affiliations:** 1 International Nursing school, Hainan Medical University, 3 Xueyuan Road, Longhua District, Haikou, China; 2 Jiangsu Health Vocational College, Nanjing, China; 3 The Second Affiliated Hospital of Hainan Medical University, Haikou, China

**Keywords:** Group care, Midwifery care, Newborn, Delivery, Self-efficacy

## Abstract

**Background:**

The group care is a well-established maternal care model that has been widely used in many developed countries, but in China, it is confined to prenatal care services. In addition, affected by traditional birth culture, Chinese women tend to focus more on their fetuses and newborns but lack attention to their own intrapartum and postpartum care. The aim of this study was to construct and implement a prenatal, intrapartum, and the postpartum continuous group care model that combines online and offline service in Hainan Province, China, and to evaluate the effect on maternal women and newborns.

**Methods:**

This study was a randomized controlled trial involving 144 pregnant women in a first-class tertiary general hospital in Hainan Province, China. Women were divided into an intervention group and a control group using the random number table, with 72 women in each group. The control group received routine maternal care services, and the intervention group received the continuous group care based on the routine maternal care services. Count data such as rate of cesarean section and incidence rate of fetal macrosomia were analyzed with the chi-square test or Fisher’s exact test, and the General Self-efficacy Scale scores were analyzed by repeated measures ANOVA. *P* < 0.05 was considered statistically significant, with two-sided probability values.

**Results:**

Compared with the control group, the rate of excessive prenatal weight gain, cesarean section, and 42-day postpartum depression were significantly lower in the intervention group (*P* < 0.05), and higher General Self-efficacy Scale scores (in the expectant period and 42 days postpartum) and exclusive breastfeeding rate (42 days postpartum) (*P* < 0.05). The incidence of fetal macrosomia was significantly lower in the intervention group (*P* < 0.05). But there was no significant difference in birth weight, preterm birth, the incidence of low-birth-weight infants and 1-min Apgar score (*P* > 0.05).

**Conclusion:**

The continuous group care with online and offline service can effectively control the gestational weight gain, reduce the rate of cesarean section, macrosomia, and postpartum depression. It can improve the self-efficacy of women and the rate of exclusive breastfeeding effectively.

**Trial registration:**

Chinese Clinical Trial Regestry (ChiCTR2200065765, 04/11/2022, Retrospectively registered).

**Supplementary Information:**

The online version contains supplementary material available at 10.1186/s12884-023-05882-1.

## Background

The global incidence of cesarean sections (CS) has been continuously rising over time. Based on data published by the World Health Organization (WHO) in 2015, the average rates of CS worldwide and across Asia stood at 19.1% and 19.2%, respectively [1]. In China, however, the prevalence of this procedure surged to as high as 36.7% in 2018 [2], which is much higher than the upper limit (15%) recommended by the WHO. Studies have shown that the main causes of the high CS rate in China are fear of childbirth pain, misunderstanding of the pros and cons of delivery modes, and lack of self-efficacy in the perinatal period [3, 4]. The essential problem underlying these causes is the inadequacy of maternal care services and health education for women. In China, the current prenatal care focus on disease and complications screening [5]. Although the government encourages hospitals with maternity departments to establish pregnancy schools in clinics, aiming to enhance health education for pregnant women and their families and improve women’s health literacy and skills, the current available health education is primarily led by healthcare providers alone without considering the individual needs of women and their families [6]. The rigid form of health education leads to the passive acceptance of women due to its lack of pertinence and individualization [7]. At the same time, the current health education system neglects the psychological and social needs of women, resulting in an information gap between healthcare providers and recipients, as well as a failure to evaluate the level of acceptance of healthcare services and improvements in women’s health issues [8–10]. Consequently, the rate of CS and postpartum depression may increase [11, 12]. Therefore, it is urgent to develop a set of effective maternal care programs that are continuous, targeted, individualized, proactive, and consistent with Chinese background. In recent years, the group care has become a focus in midwifery research. It is also known as Centering Pregnancy (CP), a pregnancy-centered healthcare model that integrates prenatal clinical visits, health education, and peer support to potentially overcome the limitations of current perinatal care models in China [13, 14].

The group care was first proposed by the midwife of Sharon Schindler Rising on the basis of a self-care project of the American College of Obstetricians and Gynecologists (ACOG) [13]. Now it has already been widely utilized in hospitals, communities and private clinics in the United States, Australia, Canada, the United Kingdom, and parts of Africa [15–19]. This model categorizes pregnant women into groups based on similar gestational age, with each group containing 8 to 12 pregnant women and their families, and incorporates prenatal visits, health education, and peer support. The emphasis lies in the need for prenatal care to prioritize pregnant women’s health education, encourage active participation in pregnancy management through diverse support from peers, midwives and family members, and foster self-efficacy via self-care and decision-making to enhance maternal and infant health [13]. A retrospective study involving 6155 women in the United States showed that compared with the traditional prenatal care, the group care resulted in more advanced gestational age (within the normal range) at childbirth, higher normal birth weight in full-term newborns, and less fetal death [20]. Tucker et al. [21] found that women who received the group care were more knowledgeable about pregnancy and had an increased sense of anticipation of labor. Heber et al. [22] demonstrated that the group care has the potential to mitigate women’s stress levels, enhance their confidence in childbirth, improve their satisfaction with prenatal care, and augment their decision-making abilities. Other researchers [23, 24] showed that women in the group care gained a sense of self-control and empowerment, acquired strength through self-management, and received more companionship and support from families. Therefore, the group care plays positive roles in promoting natural childbirth and sustaining harmonious family relations.

In recent years, several studies have tried to explore the extension of the group care into the postpartum period which focus on postpartum contraception care, infant health and development, and parenting [25, 26]. The specific group care can continue even until the child is 3 years old [26]. Although the group care has been proven effective in improving maternal and infant health, some researchers have pointed out challenges to launching the ACOG-authorized program of CP, such as licensing fees for sites, facilitator training workshops, and session materials [27]. Therefore, how to make the implementation of the group care cost-effective and flexible in diverse healthcare systems and cultures requires further investigation. In China, the group care was first introduced in 2016 [28, 29] and has been gradually explored and applied. The group care team in China generally consists of 3 to 10 midwives, obstetricians, and nurses [30]. Generally, the group care consists of three primary modules: prenatal assessments, health education, and social support [30–32]. Women are categorized based on their gestational age, with each group consisting of 8–12 women and their families. The group care team organizes 6–12 sessions during the pregnancy period, which include health education, discussions and sharing, prenatal meetings, and communication online [30–32]. Studies have shown that the group care can improve the natural delivery rate, reduce the rates of vacuum extraction, episiotomy, and neonatal asphyxia [30, 31], shorten the duration of labor, reduce maternal blood loss [32], improve the self-management ability [33], reduce the incidence rate of maternal anxiety and depression [31], and improve the breastfeeding rate [34]. However, the group care in China has paid little attention to intrapartum and postpartum care, which may be related to the culture of postpartum confinement *(zuo yue zi*) in China. It usually lasts one month after birth. During this period, mothers are confined indoors and tend to prioritize the care of their newborns over their own healthcare needs [35]. Meanwhile, there are many challenges associated with implementing face-to-face support during the COVID-19 pandemic in China, including restrictions on the companions in birth rooms due to space limitations. However, with the development and popularization of modern information technology in China, convenient online interventions may solve the problems mentioned above. Moreover, In China, midwifery care is limited to the labor ward and pregnancy school which are generally not launched until 28 gestational weeks later in Hainan Province, making it difficult to provide midwifery care during the pregnancy, especially in the first and second trimester, so the sessions of the group care began in the 30 weeks of gestation in this study. Therefore, based on the survey results of local women’s demand for the group care in Haikou (Appendix), this study developed a hybrid continuous the group care model that combines online and offline service to enhance the quality of the group care in China. The service duration covers both prenatal, natal and postnatal periods. This specific model aims to establish and implement appropriate group care practice under Chinese cultural background.

## Methods

### Study design

**Fig. 1:**
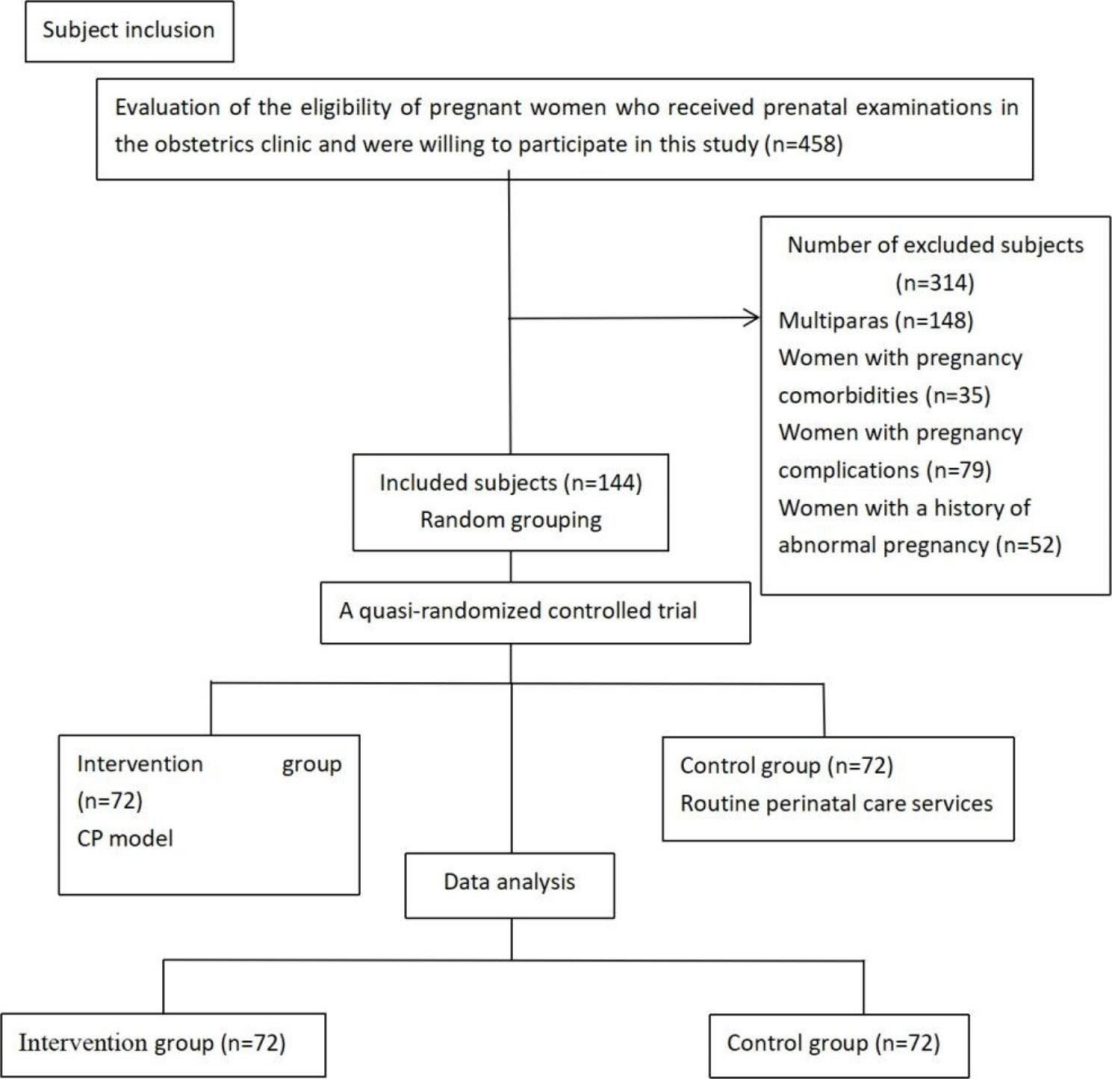
Flowchart of the study

A randomized controlled trial was conducted in this study. Women at the gestational age of 30–31 weeks who received regular prenatal examinations in the obstetrics clinic of a first-class tertiary general hospital in Hainan Province, China, from July 2020 to December 2020 were informed about the recruitment. A total of 458 showed the interest. After screening and communication, 314 subjects were excluded, including 148 multiparas, 35 women with pregnancy comorbidities, 79 women with pregnancy complications and 52 women with a history of abnormal pregnancy. Finally, 144 pregnant women at the gestational age of 30–31 weeks were enrolled in this study (see Fig. 1). The subjects were selected strictly in accordance with the inclusion and exclusion criteria and were randomly divided into an intervention group and a control group by random number table, with 72 women in each group. The general demographic data of the two groups were not significantly different (*P* > 0.05) and were balanced and comparable (Table 1).

**Table 1:** Comparison of the sociodemographic data between the intervention and control groups (N = 144)

Category		Intervention group (n = 72)	Control group (n = 72)	*t/χ* ^2^	*P*
Age (years) ($\stackrel{-}{x}$±*s*)		27.08 ± 3.03	27.10 ± 3.60	-0.05	0.96
Gravidity (number of times) ($\stackrel{-}{x}$±*s*)		1.12 ± 0.43	1.15 ± 0.39	-0.39	0.69
Gestational age (days) ($\stackrel{-}{x}$±*s*)		274.99 ± 6.27	274.40 ± 7.45	0.51	0.62
Height (cm) ($\stackrel{-}{x}$±*s*)		159.82 ± 5.12	158.22 ± 5.65	1.76	0.08
Weight (kg) *(*$\stackrel{-}{x}$±*s*)		50.11 ± 5.33	51.80 ± 5.95	-1.59	0.11
Occupation (n, %)	Farmer	3 (4.17)	4 (5.56)	1.42	0.92
	Civil servant/public institution employee	19 (26.39)	16 (22.22)		
	Private enterprise employee	16 (22.22)	13 (18.06)		
	Housewife	21 (29.17)	22 (30.56)		
	Self-employed	10 (13.89)	12 (16.67)		
	Unemployed	3 (4.17)	5 (6.94)		
Education level (n, %)	Junior high school and below	6 (8.33)	7 (9.72)	0.41	0.82
	High school and technical secondary school	17 (23.61)	14 (19.44)		
	College degree and above	49 (68.06)	51 (70.83)		

### Participants

The study participants were healthy primiparas (aged 18–35 years; 30–31 weeks of gestation) with singleton, cephalic presentation, normal fetal development, and favorable conditions for vaginal delivery. All participants were expected to give birth in the research hospital and received the health education service of the pregnancy school starting from 28 weeks, had good cognitive function, and were willing to participate in this study and to use WeChat online service during pregnancy. Their family members also voluntarily accompanied them in the sessions of the group care.

### Data collection tools

The data were mainly collected using a personal information form, a clinical observation form, the General Self-efficacy Scale (GSES), and the Chinese version of the Edinburgh Postpartum Depression Scale (EPDS).

#### Personal information form

The personal information form was developed by the researchers. The main items included the age, birthplace, education, occupation, gestational age, gravidity, prenatal weight, and body mass index (BMI) of women. The form was created in Word and printed out.

#### Clinical observation form

The clinical observation form included items such as prenatal weight gain, delivery date, delivery method, newborn weight, Apgar score and rate of 42-day exclusive breastfeeding, and so on. The form was created in Word and printed out in hard copies.

#### GSES

The GSES was developed by Schwarzer et al., and the Chinese version was translated and revised by Wang. This version has been widely used in China, with Cronbach’s α of 0.87 [36] and high reliability. The ratio of chi-square to degree of freedom is 3.92, the normative fit index is 0.96, the adjusted goodness-of-fit index is 0.93, the goodness-of-fit index is 0.95, the Tucker-Lewis index is 0.95, the comparative fit index is 0.96, and the square root of approximation error is 0.07. The scale comprises 10 items, each evaluated using a four-point scoring system, resulting in a total score range of 10 to 40 points. A higher score indicates greater levels of self-efficacy.

#### EPDS

The Chinese version of the EPDS is the most commonly used assessment tool for screening postpartum depression in China, with Cronbach’s α of 0.76 and a validity of 0.93. The scale has a total of 10 items, and each item is evaluated based on the severity of symptoms using a four-level scoring system (0 to 4 points). The presence of a total score ≥ 13 points indicates varying degrees of postpartum depression. Furthermore, a higher score corresponds to a more severe level of depression.

### Data collection

The personal information form, the GSES, and the EPDS were applied in-person. When the questionnaires were distributed, the researchers explained the purpose and precautions to the research subjects in detail by using standardized instructions, and then, the subjects completed the questionnaires by themselves. The sociodemographic data were collected at the time of enrollment of the two groups of subjects. Self-efficacy was measured before the intervention, during the expectant period, and at 42 days postpartum. Depression was measured at 1 week postpartum and 42 days postpartum. Clinical observation data were collected using the electronic information system of the hospital and were input into the clinical observation form by the researchers.

## Implementation of the continuous group care

The intervention was conducted between July and December 2020 with the COVID-19 pandemic in China. However, Hainan, being an island province, effectively curtailed exogenous transmission through timely implementation of island closure management measures, resulting in swift containment of the COVID-19 outbreak. In October 2020, there was only one additional imported case of COVID-19 due to the surge in tourism during National Day [37]. As local life returned to normal, the implementation of this study had proceeded smoothly.

### Control group

(1) In the prenatal period, routine prenatal examinations included blood pressure, body weight, uterine height, and abdominal monitoring. Fetal heart rate was monitored every 2 weeks after 32 weeks of gestation and every week after 36 weeks of gestation. The participants regularly received B-mode ultrasound and related laboratory examinations. Obstetric physicians provided prompt responses to the issues of individual women, while midwives launched collective health education to women during pregnancy check-ups (gestational week 28 and thereafter) at the pregnancy school.

(2) During the intrapartum period, due to policies for personnel management during the COVID-19, the hospital did not allow childbirth companions in labor room, women were required to enter the delivery room alone once they reached the first stage of labor. Therefore, in the first stage, the midwife mainly encouraged the family to provide support for women through voice and video calls, affirmed their independent decision regarding delivery mode and pain relief method, guided their family to prepare energy supplements such as functional drinks and chocolates, and promoted adequate nutrition intake to ensure sufficient energy during labor. In the second stage, the midwife encouraged them to actively apply breathing and pain relief techniques, and instruct them to correctly use abdominal pressure. In the third stage, wounds were examined and sutured, and the early skin-to-skin contact and early sucking were implemented.

(3) In the postpartum period, mother and baby were accommodated in the same room with close monitoring of maternal bleeding and urination within two hours after birth. Mothers were treated promptly and symptomatically if abnormalities were noted, or then they were sent back to the ward if no abnormalities were observed. Postpartum uterine involution and lochia discharge were monitored, and the mothers’ perineum were disinfected. Before discharge from the hospital, the midwife provided mothers with health education on breastfeeding and neonatal care and informed them to visit for a follow-up at 42 days postpartum.

### Intervention group

In addition to receiving the routine maternal care in the hospital, women in the intervention group were divided into 6 subgroups by similar prenatal examination times. Each subgroup consisted of 12 pregnant women and their families, and the subgroup members were fixed to form a stable intragroup relationship. Researchers promptly established WeChat online groups for each subgroup to facilitate the participation of women and their families in continuous group care.

The nursing workflow for each session of the group care was implemented in 5 steps: clarifying problems, expressing emotions, setting goals, formulating plans, and evaluating results. During implementation, midwives used different methods, such as PPT presentations, model explanations, and role-playing, to carry out health education. During each offline meeting, women and their families sat in a circle. After the midwife introduced the discussion topic, women and their families expressed their opinions on the topic and shared their own experiences, and women were encouraged to take the initiative to learn, discuss topics among themselves, and practice. In the last 20 min of the session, the midwife summarized the topic and served as a health facilitator. For those who failed to participate in offline discussions on that day, midwives provided WeChat online learning services, which could be accessed at any time, to encourage women in online WeChat groups to actively express their personal opinions, perceptions, and doubts so that women in the group could deepen ties and cultivate trust among themselves.

(1) In the prenatal period, 6 discussion topics were identified based on a survey result of local women’s needs for the group care in Haikou (see Table 2). The sessions were held at the pregnancy school of the hospital from Monday to Friday (2 h each day). On the basis of the number of prenatal examinations that the participants received in this hospital (30 weeks, 32 weeks, 34 weeks, 36 weeks, 37 weeks, and 38 weeks of gestation), a total of 6 sessions (see Table 2) were held after 30 weeks of gestation. One week before each session, notices and discussion topics were published online in the WeChat group to facilitate the participation of women and their families. Each group member should participate in at least 4 face-to-face sessions. Group members absent from face-to-face meetings should participate in another group offline or in the WeChat group online.

**Table 2:** Themes of six sessions in the continuous group care

Themes
Changes in newborns and daily nursing knowledge
Cooperation with the midwife to prevent perineal laceration
Signs of labor, preparation for labor, and prenatal abnormalities
Self-monitoring and prenatal education
Companionship methods and skills of companions and husbands during childbirth
Pregnancy nutrition

(2) During the intrapartum period, in addition to receiving standard care as provided to the control group, women in the intervention group also receive online peer support facilitated by midwives. The midwife guided other women in the same WeChat group to provide encouragement and emotional support throughout labor and maintain communication with her during this process.

(3) During the postpartum period, WeChat groups were utilized for maintaining online communication, facilitating discussions and providing guidance to women and their families. The final group session was conducted at 42 days after birth, focusing on breastfeeding and neonatal care. Subsequent to this session, continuous online discussions and communications were sustained through WeChat groups.

## Study variables

The study’s dependent variables comprise the rate of excessive weight gain in pregnancy, 1-min Apgar score, neonatal weight, low-birth-weight, cesarean section, episiotomy occurrence, macrosomia incidence, preterm birth incidence, exclusive breastfeeding duration, self-efficacy level and postpartum depression. The independent variable is the group care interventions received by women, while age, education level, occupation status, gestational age as well as prenatal weight and BMI are considered control variables.

## Data analysis

In this study, IBM SPSS 23.0 was utilized for the data analysis and processing. The sociodemographic data were presented in terms of frequencies, percentages, means, and standard deviations. They were analyzed using the independent sample *t-test* and the chi-square test to compare the age, gravidity, gestational age, weight, occupation, birthplace, and education level between the two groups. Count data such as cesarean section rate, postpartum depression incidence, breastfeeding prevalence, macrosomia occurrence, preterm birth occurrence and low-birth-weight frequency were subjected to chi-square test or Fisher’s exact probability test for analysis. Repeated measures ANOVA was employed to analyze GSES scores. The independent *t-*test was used to compare the neonatal weight and 1-min Apgar score. *P* < 0.05 was considered statistically significant, with two-sided probability values.

## Ethical considerations

This study was approved by the Ethics Committee of Hainan Medical University (Approval number: HNLL-2020-54; approval date: March 17, 2020). When the subjects were enrolled, researchers used standardized instructions to introduce the purpose, significance, and methods of this study in detail and ensured that the subjects had been informed consent regarding the entire study process, voluntarily participated in the study, and signed the hard copies of an informed consent form. The study was designed so that it would not infringe on the rights and interests of the research subjects, and the subjects could withdraw from the study at any time.

## Results

The socio-demographic data for the intervention group and control group exhibited a normal distribution. The results showed that there was no significant difference in the baseline data between the two groups (*P* > 0.05), as seen in Table 1.

For the participation of the specific group care, all women (100%) of the intervention group participated in a minimum of 4 face-to-face sessions during the prenatal period. 97.2% (n = 70) attended all 6 face-to-face group meetings during pregnancy and the final session in postpartum period, while two women attended 5 offline sessions during pregnancy and the final postpartum session. However, the two participants who missed one session were able to make up for it by attending the same topic in another group. Before the offline session, each woman discussed the session theme published by midwives in the WeChat group, with an impressive 100% participation rate. On the other hand, women exhibited the high level of engagement in online WeChat group care, particularly on the day following face-to-face group meetings. A certain number of women participated in the discussion of free themes every day, but the level of engagement was different. Very few women with zero participation. Unfortunately, the proportion of daily and individual participation online was not analyzed thoroughly.

The results showed that there was no significant difference in the rate of excessive weight gain in pregnancy, the durations of each stage of labor, the total duration of labor, the postpartum two-hour blood loss, the rate of episiotomy, the incidence rate of depression at one week postpartum, 1-min Apgar score, neonatal weight, and incidence rate of low-birth-weight infant between the two groups (*P* > 0.05). The preterm birth incidence was zero in both groups. The other indicators such as CS rate, weight gain in pregnancy, incidence rate of macrosomia, the incidence rate of depression at 42 days postpartum and exclusive breastfeeding rate 42 days postpartum were significantly different between the two groups (*P* < 0.05), as Seen in Table 3 for details.

**Table 3:** Comparison of effects between the intervention and control groups (N = 144)

Category		Intervention group (n = 72)	Control group (n = 72)	*t/χ*^2^/Z	*P*
Cesarean section rate (n, %)	Nonmedically indicated cesarean section	5 (33.33)	20 (54.05)	14.57	< 0.001
	Medically indicated cesarean section	10 (66.67)	17 (45.95)	-	-
	Total number of participants who received cesarean section	15 (20.83)	37 (51.39)	-	-
Weight gain (kg) ($\stackrel{-}{x}$±*s*)		13.40 ± 3.30	15.10 ± 4.50	-2.50	0.01
Number of participants with excessive weight gain (n, %)		12 (16.67)	28 (38.89)	8.86	0.003
Rate of excessive weight gain in different BMI ranges (n, %)	BMI (< 18.5)	2 (2.78)	4 (5.56)	-	0.68
	BMI (18.5–24.9)	10 (13.89)	24 (33.33)	7.56	0.006
	BMI (25-29.9)	0 (0)	0 (0)	-	-
	BMI (> 30)	0 (0)	0 (0)	-	-
Delivery process for participants with a natural delivery (min) M (QL–QU)	Duration of the first stage of labor	530 (325–720)	540 (370–780)	-0.86	0.39
	Duration of the second stage of labor	42 (24–62)	42 (25–60)	0.76	0.45
	Duration of the third stage of labor	8 (6–10)	8 (6–19)	-0.56	0.58
	Total duration of labor	640 (364–807)	590 (420–827)	-1.09	0.28
Postpartum 2-hour blood loss (ml) ($\stackrel{-}{x}$±*s*)		151.75 ± 56.67	190.57 ± 58.60	-3.15	0.81
Episiotomy rate (n, %)	Episiotomy	26 (45.61)	19 (54.28)	0.65	0.42
	No episiotomy	31 (54.39)	16 (45.71)	-	-
Incidence rate of macrosomia (n, %)		0 (0)	6 (8.33)	-	0.03
Postpartum EPDS score ≥ 13 (n, %)	1 week postpartum	5 (6.94)	12 (16.67)	3.27	0.07
	42 days postpartum	2 (2.78)	13 (18.05)	9.01	0.003
Exclusive breastfeeding rate 42 days postpartum (n, %)		62 (82.60)	46 (61.30)	9.48	< 0.001
Neonatal weight (kg) ($\stackrel{-}{x}$±*s*)		6.14 ± 0.60	6.38 ± 0.95	-1.76	0.08
Incidence of low-birth-weight infant(n, %)		1(1.39)	3(4.17)	-	0.62
1-min Apgar score ($\stackrel{-}{x}$±*s*)		9.94 ± 0.23	9.92 ± 0.28	0.65	0.52
Incidence of preterm birth (n, %)		0(0)	0(0)	-	-

**-**: *None*

**Fig. 2:**
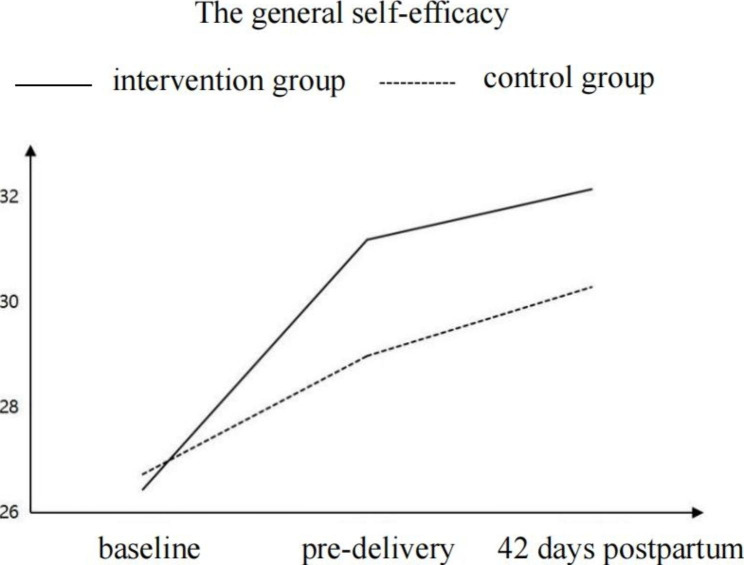
Time trend of General Self-efficacy Scale scores for the 2 groups before and after the intervention

The results showed that the GSES scores for the intervention group and control group increased with the extension of the intervention time, but the increasing trend for the intervention group was more significant (*P* < 0.05), as shown in Table 4; Fig. 2.

**Table 4:** Comparison of the General Self-efficacy Scale scores between the 2 groups (N = 144)

Group	At the time of enrollment/points *(*$\stackrel{-}{x}$±*s*)	Before childbirth/points ($\stackrel{-}{x}$±*s*)	42 days postpartum /points ($\stackrel{-}{x}$±*s*)	Time main effect	Main effect of intervention	Interaction Effect
				*F* (*P*)	*F* (*P*)	*F* (*P*)
Intervention group (n = 72)	26.44 ± 3.55	31.19 ± 3.51	32.15 ± 3.12	48.96	11.44	3.95
Control group (n = 72)	26.73 ± 5.58	28.98 ± 1.89	30.29 ± 2.71	< 0 .001	0.001	0.02
*t*	-0.37	4.69	3.82	-	-	-
*P*	0.71	< 0.001	< 0.001	-	-	-

**-**: *None*

## Discussion

This is the first study in China to integrate online and offline services, covering prenatal, natal, and postnatal periods within group care. The findings demonstrated the reduction of CS, excessive prenatal weight gain, postpartum depression, fetal macrosomia in women who participated in the continuous group care combined online and offline service. It also showed the significant increase in GSES scores and the rate of exclusive breastfeeding over the same period when compared to the control group. Furthermore, women in the intervention group demonstrated a high level of participation.

In this study, the intervention group demonstrated a higher participation in the face-to-face sessions compared to clinic norms (50-60%) and previous studies [38, 39], indicating strong potential for generalizability within the local region. It was considered that the high participation may be attributed to several factors, with the primary reason being the intrinsic motivation of women to acquire knowledge and skills related to prenatal health [40]. In this study, researchers tailored the intervention based on a needs assessment survey conducted among local women (Appendix), which ensured that the discussion topics in sessions were relevant and engaging for participants. Secondly, the educational level of the most women is high (college degree and above, 68.06%), which also makes them more receptive to the sessions. Furthermore, the study groups held 6 offline sessions (beginning in weeks 30–31), which is a shorter duration than Centering Pregnancy’s 8 to 10 sessions (beginning in the second trimester) [41]. Due to the short duration and fewer sessions, participants may exhibit greater engagement and adherence. However, the absence of sessions in the second trimester due to local obstetric management policies and midwives’ occupational limitations in China highlights the need for government support in reforming midwifery practice.

The findings indicated that the intervention group exhibited a significantly high level of engagement with WeChat online services, which may be attributed to the impact of the COVID-19 pandemic [42, 43] and traditional postpartum practices known as *“zuo yue zi”* [44]. Online services offer greater convenience, accessibility, and flexibility for women seeking continuous help during the prenatal, natal and postnatal period. Moreover, they represent a safer alternative approach during critical periods such as the COVID-19 pandemic [45]. Additionally, the pre-intervention survey revealed that there was a significant demand among women for breastfeeding (85.00%), neonatal care (95.00%), and postpartum pelvic floor rehabilitation (84.70%) (see Table S2 in Appendix). As a result, there has been sustained high online participation during postpartum periods. However, while this study demonstrated high online participation, researchers did not thoroughly calculate the degree of online activity and participation rate. Therefore, further exploration is necessary in future research.

Studies show that the lack of self-efficacy is one of the main reasons caused the increase in the rate of nonmedically indicated cesarean Sect. [29]. In this study, the level of general self-efficacy of women who participated in the specific group care was higher than that of the control group before childbirth and at 42 days postpartum, and the increasing trend of self-efficacy continued over time, this is consistent with the results of Hu et al. [46]. In each group, the gestational age and expected birth date of the women were similar, so they had similar problems with pregnancy and childbirth. The peer support as the main characteristic of the group care provided by both offline and online in this study enables participants to engage in discussions about their shared challenges and effectively express their emotions, thereby enhancing their ability to cope with the unique difficulties at each stage [13]. Additionally, this support from group facilitates the acquisition of relevant knowledge and skills, ultimately leading to improved self-efficacy during labor [47]. Meanwhile, the themes of six sessions in the study are from the survey of local women’s demands about group care, midwives can help women to fully understand the different modes of delivery and the adverse effects of CS through online and offline group discussions. Consequently, women actively managed their diet and exercise during pregnancy to create favorable conditions for natural birth, thereby it may reduce the CS rate. Furthermore, although there is no family companion in the labor room because of policies for personnel management during the COVID-19 pandemic, online group care provided encouragement and emotional support throughout labor. The woman may feel the companionship and support from peers online to enhance their confidence in natural childbirth. Therefore, the continuous group care with online and offline service is likely to be an effective, and feasible model for improving women’s self-efficacy, and reducing the rate of CS in China.

Although several studies evaluated the effect of the group care on the gestational weight gain of women have inconsistent results [47]. The finding of this study showed that the continuous group care can effectively help women control excessive prenatal weight gain, and reduce the incidence rate of fetal macrosomia. Through participation in sessions, women developed a more accurate understanding of pregnancy nutrition and the significance of weight management in promoting both their own well-being as well as that of fetuses. Midwives also help women to measure their body weight every day so that they could monitor their own weight gain and fetal weight gain during pregnancy. Meanwhile, midwives help the women to choose various forms of exercise that met the individual needs of each woman by online and offline service. Moreover, women who achieve appropriate weight gain serve as positive role models and encourage the entire group to proactively develop a sensible weight management plan during pregnancy. They adopt various effective dietary and exercise strategies to regulate their own weight gain and fetal growth, thereby effectively preventing the occurrence of fetal macrosomia [48]. Liu et al. [49] demonstrated that higher levels of self-efficacy among women are associated with better weight control during pregnancy. This finding is consistent with the research by Liu et al. [49] and may be attributed to the use of group care, which helps women develop a correct understanding of nutrition and fetal weight control, as well as establish confidence in self-care. As a result, personalized assessments can be made and successful management of both maternal and fetal weight achieved.

In this study, there was no significant difference in the duration of each stage of labor or the total duration of labor between the two groups (*P* > 0.05), a result that is different from the finding by Guo et al. [50] that the application of the group care in women with a second pregnancy at advanced maternal age shortened the duration of each stage of labor and the total duration of labor. The reasons for this discrepancy might be that the subjects of this study were all primiparas, whose cervix had never been dilated; alternatively, the duration of labor could have been affected by many factors, such as the women’s physiological conditions and emotions. Some previous studies show that family companions can alleviate the labor pain and reduce the duration of labor effectively [51, 52]. In this study the hospital did not allow companions under the background of the COVID-19 pandemic. Therefore, women had to enter the delivery room alone, so the family member could not support women through direct touch or face-to-face communication during the labor. Despite the availability of peer support through the WeChat group, factors such as lacking of family companion and labor pain may mitigate effects of emotional information support via mobile phone, resulting in no significant difference in labor duration between the two groups. However, it demonstrates another area of research interest may emerge, focusing on the impact of a combined intervention involving birth companions and online group care on labor duration in the post-COVID-19 pandemic.

This study showed that the postpartum 2-hour blood loss in the intervention group was less but not significant than that of the control group(*P* > 0.05), being consistent with the results of Hu et al. [46]. This may be related to the fact that the participants in this study were all healthy primiparas with no abnormal pregnancy history and possibly related to the fact that maternal blood loss was measured visually and not through a more accurate method. Meanwhile, the result showed that there was no significant difference in the episiotomy rate between the two groups (*P* > 0 0.05) and the episiotomy rate of the intervention group (45.61%) and control group (54.28%) were both higher than other countries [53], that may be related to the fact that the hospital in this study did not strictly implement episiotomy. This finding is consistent with the study by Wang et al. [31]. Because there is no uniform standard for episiotomy in China, under normal circumstances, midwives decide whether to perform episiotomy based on empirical judgment [54]. A previous study demonstrated that the risk of episiotomy was 10.3 times higher among women with poor perineal elasticity, as determined by empirical judgment alone, compared to those with average elasticity. Additionally, the risk of episiotomy was found to be 730.82 times greater among women with an estimated fetal weight ≥ 4000 g than those with an estimated fetal weight < 3000 g [55]. Although women have discussed the decision-making to critically accept episiotomy during childbirth in the session, midwives and obstetricians are still in authoritative positions in childbirth services in China, especially in economically underdeveloped areas [56], leading to the weakening of women’s autonomy and the loss of their right to participate in decision-making regarding childbirth. Therefore, in the future studies, it is imperative that midwives undergo standardized training beforehand and utilize scientific tools to avoid subjective empirical practices and minimize data bias resulting from inter-midwife differences. Moreover, further exploration requires the establishment of scientific evaluation criteria for episiotomy and the enhancement of women’s autonomy and decision-making during childbirth.

Worldwide, postpartum depression affects approximately 10–15% of women, and the incidence rate in China has reached 15–30% in recent years [57, 58]. In this study, there was a significant difference in the EPDS scores 42-day postpartum between the two groups; that is, the group care effectively reduced the incidence rate of postpartum depression. Prenatal anxiety is one of the strongest risk factors for postpartum depression [59]. After members of subgroups fully establish a trusting peer relationship in this study, they can relieve their anxiety by confiding in the group, sharing their psychological feelings, and gaining understanding and emotional support. It is different from other studies that the group care of this study was continuously applied up to 42 days postpartum [21]. Women were able to maintain both online and offline discussions and communication before and after childbirth. Evidently, the continuous group care can establish peer relationships during pregnancy that last postpartum. This long-established trust relationship allows women to take the initiative to solve and cope with various discomforts during the puerperium, and improve their psychological status, all of which may reduce the rate of postpartum depression.

A survey of parental demands with regard to the group care found that parents have the highest level of demands for neonatal care [51]. Studies have shown that the group care model can promote women’s exclusive breastfeeding behavior because parents’ decision-making regarding infant feeding occurs before childbirth and is influenced by positive attitudes and peer support in the group [50]. This study also demonstrated a significant increase in exclusive breastfeeding rates at 42 days postpartum among the intervention group (*P* < 0.05). With the continuous group care covering prenatal, natal, and postnatal periods, women not only learn about the benefits of breastfeeding over formula feeding and express their opinions on neonatal feeding during sessions and online WeChat groups but also share their new experiences and challenges about breastfeeding online during the postpartum period. Through the continuous group discussions and the exchange of experiences, women in the same group established attitudes toward exclusive breastfeeding and mastered the skills of exclusive breastfeeding successfully. However, whether the continuous group care is more effective than the prenatal group care in improving breastfeeding needs further study.

For the neonatal outcomes, the Apgar score is widely used as a clinical index to assess the health of newborns. The result of this study indicated that there was no significant difference in the two groups (*P* > 0.05), which is consistent with the previous studies conducted by Hu [46] and Wang [31]. This similarity may be attributed to the subjective nature of the Apgar score and the fact that most women in both groups underwent the high rate of episiotomy to expedite fetal delivery, resulting in a relatively short second stage of labor and lower possibilities of fetal distress. Moreover, this study showed that there was no significant difference in the incidence of birth weight, low-birth-weight infants and preterm birth between the two groups (*P* > 0.05). However, a Meta-analysis showed that the group care was associated with a decreased rate of low-birth-weight and there is no significant difference in the rate of preterm birth [47]. The different results may be due to the small sample size in this study. In the future, large studies should be needed to further investigate the effect of the continuous group care on the neonatal outcomes in China.

Compared to the group care in other studies, the continuous group care with online and offline services begins at 30 weeks with 6 prenatal sessions and 1 postnatal session, rather than starting in the second trimester with 8 to 10 prenatal sessions [41]. However, the majority of the findings in this study regarding maternal and neonatal outcomes are consistent with those of other studies [47, 50]. The 100% participation of the study in prenatal and postnatal sessions may be a key factor. Additionally, this specific group care model incorporates the philosophy of continuous midwifery care advocated by ICM [60] and effectively utilizes online services to adapt to the COVID-19 pandemic and unique postpartum culture in China. But in fact, it also demonstrated that the urgent demand and enthusiasm among women fort the group care in economically underdeveloped regions of China.

## Limitations

This study was conducted between July and December 2020 amidst the COVID-19 pandemic in China. Despite Haikou city experiencing minimal impact from the pandemic during this period, some women expressed concerns regarding their safety in crowded settings and were hesitant to participate in face-to-face sessions. Therefore, the process of subject recruitment posed numerous challenges for researchers, however, ultimately the majority of college-educated women demonstrated a willingness to participate in the program. Further investigation is required to determine the impact of the continuous group care on women with varying levels of education. On the other hand, as this study excluded women with multiple pregnancies and pregnancy complications in the recruitment, further research and discussion are needed to determine the applicability and effectiveness of this study for high-risk women. In addition, the continuous group care implemented in this study begins at the 30 weeks of gestation due to local obstetric management policies and midwives’ occupational limitations in China, setting it apart from other studies that initiate group care during the second trimester [41]. Further investigation is needed to determine whether this timing affects the effectiveness of group care.

## Conclusion and recommendations

In this study, we implemented the online and offline hybrid continuous group care in China; the program was significantly effective in controlling the gestational weight gain, reducing the incidence rate of fetal macrosomia, improving the self-efficacy of women, reducing CS rate, exclusive breastfeeding rate and the incidence rate of postpartum depression. This study holds significant reference value for the continued implementation of the group care model in Chinese healthcare system. Additionally, it contributes to expanding the scope of group care. It provides a reference for the extensive implementation of the group care for women in other regions of China or in some regions of other countries, and it need to take into account the needs about the group care of women, the allocation of human resources among care providers and standardized training first when conduct the group care model.

### Electronic supplementary material

Below is the link to the electronic supplementary material.

Supplementary Material 1

## Data Availability

The datasets generated and/or analyzed during the current research are not publicly available as individual privacy could be compromised but are available from the corresponding author on reasonable request.

## References

[1] World Health Organization Human Reproduction Programme A (2015). WHO Statement on caesarean section rates. Reprod Health Matters.

[2] National Health Commission of the People’s Republic of China. Report on the Development of Maternal and Child Health in China [Internet]. Available from: http://www.nhc.gov.cn/fys/ptpxw/201905/bbd8e2134a7e47958c5c9ef032e1dfa2.shtml.

[3] Wang J, Yan M, Wang L (2016). Investigation on the repeat pregnant women’s knowledge of delivery mode after cesarean section. Chin J Practical Nurs.

[4] Lei X. The application of the PRECEDE-PROCEED model in the puerperal care of unipara. University of South China; 2020.

[5] Su J (2019). Research progress of early prenatal ultrasound screening and diagnosis. China Med Device Informa.

[6] Xu Y, Yang Y, Qiu C (2020). Research progress on management model of health education for pregnant women in school. Chin J health Educ.

[7] Liu Y, Wang Y, Wu Y (2021). Effectiveness of the centering pregnancy program on maternal and birth outcomes: a systematic review and meta-analysis. Int J Nurs Stud.

[8] Hui Y, Zhang L, Li N (2020). Influence of the combination of QQ communication platform and pregnant Women School on the rate of breast-feeding and the self-care ability. Guide of China Medicine.

[9] Dai Y, Chen X, Lin J (2020). Effect of Learning Team Model combined with online courses of pregnant Women School in Training for New Nurses in Department of Obstetrics. J Nurs.

[10] Liang G, Xiao X, Zhang Q, Lin L (2019). Study on the influence of APP-based mobile information technology on reducing the fear of delivery among elderly pregnant women. J Qiqihar Univ Med.

[11] O’Neill P, Cycon A, Friedman L (2019). Seeking social support and postpartum depression: a pilot retrospective study of perceived changes. Midwifery.

[12] Betrán AP, Temmerman M, Kingdon C, Mohiddin A, Opiyo N, Torloni MR (2018). Interventions to reduce unnecessary caesarean sections in healthy women and babies. Lancet.

[13] Rising SS (1998). Centering pregnancy. An interdisciplinary model of empowerment. J nurse-midwifery.

[14] Lewis JB, Cunningham SD, Shabanova V, Hassan SS, Magriples U, Rodriguez MG, Ickovics JR (2021). Group prenatal care and improved birth outcomes: results from a type 1 hybrid effectiveness-implementation study. Prev Med.

[15] Robinson K, Garnier-Villarreal M, Hanson L (2018). Effectiveness of centering pregnancy on breast-feeding initiation among African Americans: a systematic review and Meta-analysis. J Perinat Neonatal Nurs.

[16] Allen J, Kildea S, Stapleton H (2015). How does group antenatal care function within a caseload midwifery model? A critical ethnographic analysis. Midwifery.

[17] Tilden EL, Hersh SR, Emeis CL, Weinstein SR, Caughey AB (2014). Group prenatal care: review of outcomes and recommendations for model implementation. Obstet Gynecol Surv.

[18] Liese KL, Kapito E, Chirwa E, Liu L, Mei X, Norr KF, Patil CL (2021). Impact of group prenatal care on key prenatal services and educational topics in Malawi and Tanzania. Int J Gynaecol Obstet.

[19] Eluwa GI, Adebajo SB, Torpey K, Shittu O, Abdu-Aguye S, Pearlman D (2018). The effects of centering pregnancy on maternal and fetal outcomes in northern Nigeria; a prospective cohort analysis. BMC Pregnancy Childbirth.

[20] Tanner-Smith EE, Steinka-Fry KT, Lipsey MW (2014). The effects of centering pregnancy group prenatal care on gestational age, birth weight, and fetal demise. Matern Child Health J.

[21] Tucker CM, Felder TM, Dail RB, Lyndon A, Allen KC (2021). Group prenatal care and maternal outcomes: a scoping review. MCN Am J Matern Child Nurs.

[22] Heberlein EC, Picklesimer AH, Billings DL, Covington-Kolb S, Farber N, Frongillo EA (2016). The comparative effects of group prenatal care on psychosocial outcomes. Arch Women Ment Health.

[23] Hale N, Picklesimer AH, Billings DL, Covington-Kolb S (2014). The impact of centering pregnancy group prenatal care on postpartum family planning. Am J Obstet Gynecol.

[24] Zhang S, Zhou L, Shi L. Influence of group health care model during pregnancy on midwifery based primipara′s self-efficacy and quality of lifes. Chin J Health Manage 2021,15(5):459–63.

[25] Heberlein E, Smith J, Willis C, Hall W, Covington-Kolb S, Crockett A (2020). The effects of centering pregnancy group prenatal care on postpartum visit attendance and contraception use. Contraception.

[26] Novick G, Womack JA, Sadler LS (2020). Beyond implementation: sustaining group prenatal care and Group Well-Child Care. J Midwifery Womens Health.

[27] Kettrey HH, Steinka-Fry KT (2020). Effects of March of Dimes supportive pregnancy care on maternal and Infant Health Across Diverse patient populations: a quasi-experimental multi-site pilot study. Prev Sci.

[28] Pan X, Zhang J, Zhang X (2016). Research progress in the application of foreign centering pregnancy model. Chin J Nurs.

[29] Cui P, Guo N, Wu W (2016). The current status of the Group Care Model in pregnant and maternal health and the enlightenment to mainland China. Chin Nurs Manage.

[30] Xu Y, Chu Y, Zhang L (2021). Study on the effect of group pregnancy weight management model based on ITHBC theory on primiparas of advanced age. Chin J Women Child Health.

[31] Wang J, Yan M, Liu Y (2019). Influencing of group prenatal care intervention model on pregnancy outcome. Chin Nurs Res.

[32] Zhu F, Li Q, Ding L, Xie L (2020). Effect of group-type maternal health model on maternal delivery outcomes. Chin Community Doctors.

[33] Zhou L, Zhang J, Chen D, Zhang X (2019). The application and effects of family-centered group prenatal care model. Chin J Nurs.

[34] Liao C (2019). Self-efficacy-based centering pregnancy care model on breastfeeding after caesarean section. Nurs Pract Res.

[35] Wen X, Zhai J, Xiong A (2021). The development status and enlightenment of Group Care Global of Centering-Based maternal and infant health care. Chin Nurs Management.

[36] Liu C. The relationship between fertility-related stress and quality of life among women with infertility. China Medical University; 2020.

[37] Health Commission of Hainan Province. Real-time updates of the novel coronavirus pneumonia outbreak in Hainan Province. (2020-10-21) [2020-02-25]. https://wst.hainan.gov.cn/swjw/rdzt/yqfk/202010/t20201021_2869102.html.

[38] Cunningham SD, Grilo S, Lewis JB (2017). Group prenatal care attendance: determinants and relationship with care satisfaction. Matern Child Health J.

[39] Massey Z, Rising SS, Ickovics J (2006). CenteringPregnancy group prenatal care: promoting relationship-centered care. J Obstet Gynecol Neonatal Nurs.

[40] Shi Y, Wang D, Yuan Y, Jiang Y, Zeng Q, Chang C (2015). The effect of prenatal education curriculum on mother’s prenatal examination utilization, delivery mode and recovery status: a cross-sectional survey in China. Environ Health Prev Med.

[41] DeCesare JZ, Jackson JR (2015). Centering pregnancy: practical tips for your practice. Arch Gynecol Obstet.

[42] Basu A, Kim HH, Basaldua R (2021). A cross-national study of factors associated with women’s perinatal mental health and wellbeing during the COVID-19 pandemic. PLoS ONE.

[43] Wu H, Sun W, Huang X (2020). Online Antenatal Care during the COVID-19 pandemic: Opportunities and Challenges. J Med Internet Res.

[44] Wan EY, Moyer CA, Harlow SD, Fan Z, Jie Y, Yang H (2009). Postpartum depression and traditional postpartum care in China: role of zuoyuezi. Int J Gynaecol Obstet.

[45] Uludağ E, Serçekuş P, Vardar O, Özkan S, Alataş SE (2022). Effects of online antenatal education on worries about labour, fear of childbirth, preparedness for labour and fear of covid-19 during the covid-19 pandemic: a single-blind randomised controlled study. Midwifery.

[46] Hu J, Zhou L, Tu M (2020). Influence of midwife led group health care mode during pregnancy on self-efficacy, delivery outcome and psychological state of primipara. J Nurses Train.

[47] Carter EB, Temming LA, Akin J, Fowler S, Macones GA, Colditz GA, Tuuli MG (2016). Group prenatal care compared with traditional prenatal care: a systematic review and Meta-analysis. Obstet Gynecol.

[48] Kominiarek MA, Gray EL, Vyhmeister H, Grobman W, Simon M (2018). Association of Gestational Weight Gain with prenatal care model. J Midwifery Womens Health.

[49] Liu W, Luan Z, Gong D, Wei S, Xu Q (2020). Investigation on influencing factors of self-management of pregnancy weight and general self-efficacy of pregnant women in community[J]. Chin J Gen Pract.

[50] Guo S, Ma Y, Mao X (2020). Effect of center group pregnancy care mode on outcome of delivery in elderly pregnant parturient for a second child. J Third Military Med Univ.

[51] Wang M, Song Q, Xu J (2018). Continuous support during labour in childbirth: a cross-sectional study in a university teaching hospital in Shanghai, China. BMC Pregnancy Childbirth.

[52] Bohren MA, Hofmeyr GJ, Sakala C, Fukuzawa RK, Cuthbert A. Continuous support for women during childbirth. Cochrane Database of Systematic Reviews; 2017. 10.1002/14651858.CD003766.pub6 PMC6483123 28681500

[53] Clesse C, Lighezzolo-Alnot J, De Lavergne S, Hamlin S, Scheffler M (2019). Socio-historical evolution of the episiotomy practice: a literature review. Women Health.

[54] Song K. Analysis of clinical characteristics and establishment of prediction model of lateral episiotomy in primiparas. Lanzhou Univ. 2021.

[55] Guo L, Ding Y (2018). Impact evaluation of comprehensive assessment system application during childbirth on the outcomes of perineum protection. J Nurs Sci.

[56] Li Q, Sun F, Yan T, Liu X, Sun Z (2019). Correlation between occupational stress and job burnout of midwives. China maternal and child health care.

[57] Brummelte S, Galea LA (2016). Postpartum depression: etiology, treatment and consequences for maternal care. Horm Behav.

[58] Wang Y, Zhou X, Piao D, Li T (2019). The efficacy of the combined use of cognitive therapy and collaborative nursing on patients with postpartum depression. Zhejiang Med J.

[59] Osborne LM, Voegtline K, Standeven LR, Sundel B, Pangtey M, Hantsoo L (2021). High worry in pregnancy predicts postpartum depression. J Affect Disord.

[60] The International Confederation of Midwives. Philosophy and Model of Midwifery Care. [Internet]. Available from: https://www.internationalmidwives.org/our-work/policy-and-practice/philosophy-and-model-of-midwifery-care.html.

