# Supplementary material for: Effects of the online and offline hybrid continuous group care on maternal and infant health: a randomized controlled trial
Source: BMC Pregnancy Childbirth. 2023 Sep 1;23:629. doi: 10.1186/s12884-023-05882-1 (PMC10472587; doi:10.1186/s12884-023-05882-1)
Supplement: Supplementary file 1 — Supplementary Material 1 [file 12884_2023_5882_MOESM1_ESM.docx]

**Demand analysis for the group care from pregnant women in Haikou City**

In this study, convenience sampling was used to select 325 pregnant women with natural birth experience in the obstetrics department of five hospitals from April to June 2020 in the first and Second Affiliated hospitals of Hainan Medical College, Haikou Maternal and Child Health Hospital, Hainan Hospital of Traditional Chinese Medicine, and Hainan Maternal and Child Health Hospital. 325 questionnaires were distributed, and 300 valid questionnaires were collected, with an effective recovery rate of 92.30%.

1. **Survey content**

This research includes the following aspects: (1) Socio-demographic information: including age, education, pregnancy, birth, occupation, education level, and per capita household income. (2) the group care for pregnant women in Haikou City include various aspects: prenatal environment such as group discussion, nutrition and exercise during pregnancy, signs of labor, prenatal preparation, prenatal abnormalities, non-drug analgesia, self-monitoring, and fetal education, as well as prevention of perineal laceration. Additionally, online encouragement and support for pregnant women in the group are provided during delivery. Postpartum services cover postpartum physiological recovery, pelvic floor muscle rehabilitation, breastfeeding, and newborn care.

1. **Statistical analysis**

Data was analyzed by SPSS IBM 23.0 software. Descriptive statistics of frequency and percentage were used to describe the participants’ socio-demographic and demand for group perinatal care.

1. **Results**
   1. **Socio-demographic characteristics**

A descriptive analysis was made of the socio-demographic data of 300 pregnant women, including age, education level, occupation and per capita monthly family income, as shown in **Table S1**.

**Table S1** Basic information of pregnant women

| Characteristics | n=300 |
| --- | --- |
| Age (Years) |  |
| 19~25 | 47(15.67%) |
| 26~35 | 242(80.67%) |
| 36~40 | 11(3.67%) |
| Education |  |
| Junior high school and below | 84(28.00%) |
| High school and secondary school and below | 79(26.33%) |
| Junior college and above |  |
| Job |  |
| Farmer | 8(2.66%) |
| Civil servant/public institution employee | 85(28.33%) |
| Private enterprise employee | 51(17.00%) |
| Housewife | 86(28.67%) |
| Self-employed/freelance | 48(16.00% |
| Unemployment/waiting for employment | 22(7.33%) |
| Income (CNY) |  |
| ≤1000 | 88(29.33%) |
| 1001~3000 | 61(20.33%) |
| 3001~6000 | 104(34.67%) |
| 6001~10000 | 39(13.00%) |
| >10001 | 8(2.67%) |

- 1. **Current situation of demand for the group care on women in Haikou City**

The level of demand for prenatal, postpartum, and neonatal care services were evaluated based on the responses collected from the questionnaire. The degree of demand for each category was determined by the proportion of respondents who selected “very necessary” or “necessary”. The questionnaire involved options rated from one to five, with three being considered average.

The most in-demand topics included discussions with mothers in both online and offline groups to learn about newborn changes and daily care (95%), discussions with pregnant women in the group on promoting pain-free delivery and preventing perineal lacerations (93.3%), identifying the signs of labor, prenatal preparation, and prenatal abnormalities (91.3%), the theme of self-monitoring and accompanying pregnant fathers (88.3%), the theme of diet during pregnancy (87.7%), and the theme of exercise during pregnancy (87%).

**Table S2** Order of demand for the group care services (n=300)

| Demand Content | n=300 | Order |
| --- | --- | --- |
| Discussion and learning about changes in their newborn and daily care online and offline | 285 (95.00%) | 1 |
| Discussion with women in the group about cooperating with midwives to prevent perineal laceration | 280 (93.30%) | 2 |
| Discussion about labor signs, labor preparation, and prenatal abnormalities with pregnant women in the group | 274 (91.30%) | 3 |
| Discussion about self-monitoring and fetal education with women in the group | 265 (88.30%) | 4 |
| Discussion with women in the group about the methods and skills of accompanying partners and pregnant fathers during childbirth | 265 (88.30%) | 5 |
| Discussion and learning about nutrition during pregnancy with women in the group | 263 (87.70%) | 6 |
| Discussion and learning from peers in the group who exercise during pregnancy | 261 (87.00%) | 7 |
| Discussion with women in the group to learn ways to promote comfortable delivery | 260 (86.70%) | 8 |
| Breastfeeding knowledge discussions with women in the group by online and offline | 255 (85.00%) | 9 |
| Discussion with women to learn the knowledge of postpartum pelvic floor rehabilitation through online and offline | 254 (84.70%) | 10 |
| Discussion with women in the group on WeChat postpartum about physiological recovery | 254 (84.60%) | 11 |
| Learning and discussion about pregnancy problems and confusion with women in a welcoming environment (e. g. floor mats, aromatherapy, music, multimedia, etc.) | 245 (81.60%) | 12 |
| Discussion with women in the group about different delivery methods and their advantages and disadvantages | 214 (71.40%) | 13 |
| Expectation for family members to accompany each group session | 204 (68.00%) | 14 |
| Discussion and sharing the psychological and emotional adjustment during pregnancy with women in the group | 178 (59.30%) | 15 |
| Conducting online and offline discussions with peers on postpartum emotional and psychological adjustment in the group | 176 (58.70%) | 16 |
| Encouragement, support, and company from each other on WeChat throughout the delivery process | 158 (52.60%) | 17 |
